# Type IV Pilus Assembly Proficiency and Dynamics Influence Pilin Subunit Phospho-Form Macro- and Microheterogeneity in *Neisseria gonorrhoeae*

**DOI:** 10.1371/journal.pone.0096419

**Published:** 2014-05-05

**Authors:** Åshild Vik, Jan Haug Anonsen, Finn Erik Aas, Finn Terje Hegge, Norbert Roos, Michael Koomey, Marina Aspholm

**Affiliations:** 1 Centre for Molecular Biology and Neuroscience, University of Oslo, Oslo, Norway; 2 Department of Biosciences, University of Oslo, Oslo, Norway; University of Würzburg, Germany

## Abstract

The PilE pilin subunit protein of the gonococcal Type IV pilus (Tfp) colonization factor undergoes multisite, covalent modification with the zwitterionic phospho-form modification phosphoethanolamine (PE). In a mutant lacking the pilin-like PilV protein however, PilE is modified with a mixture of PE and phosphocholine (PC). Moreover, intrastrain variation of PilE PC modification levels have been observed in backgrounds that constitutively express PptA (the protein phospho-form transferase A) required for both PE and PC modification. The molecular basis underlying phospho-form microheterogeneity in these instances remains poorly defined. Here, we examined the effects of mutations at numerous loci that disrupt or perturb Tfp assembly and observed that these mutants phenocopy the *pilV* mutant vis a vis phospho-form modification status. Thus, PC modification appears to be directly or indirectly responsive to the efficacy of pilin subunit interactions. Despite the complexity of contributing factors identified here, the data favor a model in which increased retention in the inner membrane may act as a key signal in altering phospho-form modification. These results also provide an alternative explanation for the variation in PilE PC levels observed previously and that has been assumed to be due to phase variation of *pptA*. Moreover, mass spectrometry revealed evidence for mono- and di-methylated forms of PE attached to PilE in mutants deficient in pilus assembly, directly implicating a methyltransferase-based pathway for PC synthesis in *N. gonorrhoeae*.

## Introduction

In all living organisms, many newly synthesized proteins are post-translationally modified to attain their intended function. These post-translational modifications (PTMs) involve both breakage of covalent bonds (i.e. by proteolytic cleavage) and formation of new bonds by addition of chemical moieties to the protein. Chemical groups added to the protein range from simple phosphate groups to lipids and complex polysaccharides. As a result the protein attains novel biochemical properties that can lead for instance to modulation of enzymatic activity, export, association with the lipid bilayer and targeting for or protection from degradation.

**Figure 1:**
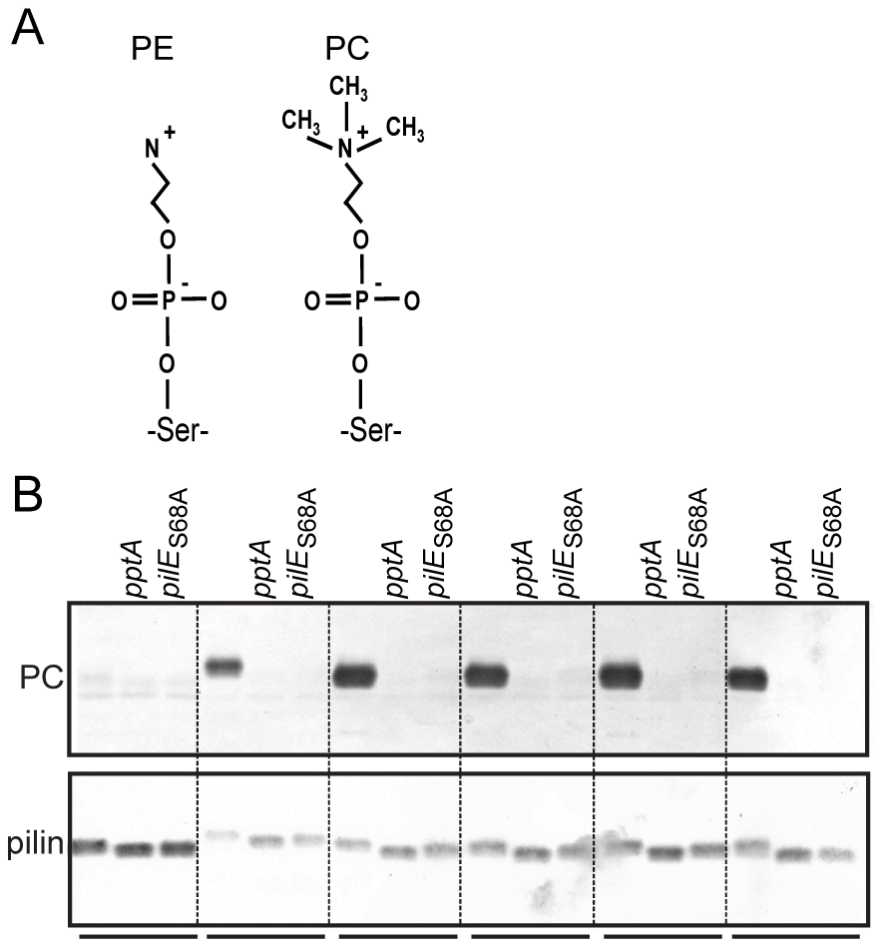
Disrupting pilus biogenesis results in PptA-dependent PC-modification of PilE. A) Schematic representation of the PE and PC structures covalently bound by *O*-linkage to serine residues of pilin. B) Immunoblots of whole cell lysates made from equal numbers of cells and of equal amounts of protein from purified pili using the PC recognizing antibody TEPC-15 (upper panel) and the PilE peptide specific α-pilin antibody (lower panel). Strains used were wild-type (N400), *pptA* (KS9), *pilE*_S68A_ (KS640), *pilD* (KS641), *pilD pptA* (KS662), *pilD pilE*_S68A_ (KS667), *pilF* (KS643), *pilF pptA* (KS663), *pilF pilE*_S68A_ (KS668), *pilQ* (KS644), *pilQ pptA* (KS664), *pilQ pilE*_S68A_ (KS669), *pilP* (KS665), *pilP pptA* (KS666), *pilP pilE*_S68A_ (KS670), *pilG* (KS674), *pilG pptA* (KS673), and *pilG pilE*_S68A_ (KS672). All samples were run on the same gel and the dotted lines were introduced as guidance facilitating evaluation of the data. Results representative of at least three different experiments are shown.

The structurally related zwitterionic moieties phosphoethanolamine (PE) and phosphocholine (PC) (here collectively referred to as phospho-form modifications) are common cell surfaces constituents of pathogenic and commensal bacteria of humans (Fig. 1A). In many Gram-negative species, phospho-form modifications are found on lipopolysaccharides and lipooligosaccharides where they influence structure, antigenicity and interactions with innate immune components [1]–[5]. It has been shown that PC modified constituents expressed on Gram-negative bacterial surfaces have the ability to impact adherence to epithelial cells via interaction with the platelet activating factor (PAF) receptor as well as to act as ligands for C-reactive protein and PC-recognizing antibodies [5]–[9]. In addition, PE and PC moieties have been identified as direct post-translational modifications of bacterial proteins. Covalent attachment of PE and PC to protein was first demonstrated in *Neisseria gonorrhoeae* where they were shown to be *O*-linked to the type IV pilus (Tfp) subunit protein PilE via a serine residue [10]. As seen by reactivity with the TEPC-15 monoclonal antibody recognizing a PC-dependent epitope, the related meningococcal pilins also undergo PC modification [11]–[13]. Recently, the lipoproteins Ngo1043 and Ngo1237 were identified as additional phospho-form modified proteins in *N. gonorrhoeae* [14]. However, the general significance of PE and PC phospho-form modifications of neisserial proteins has yet to be determined. Phospho-form modification of bacterial protein has also been described in *C. jejuni*, where the flagellar rod protein FlgG undergoes PE modification and mutants lacking this PTM display defects in flagellar expression and thus motility and virulence [15], [16].

In light of their broad distribution and potential biological significance, increased attention has been directed at characterizing the biosynthesis of phospho-form modifications. The *Escherichia coli* EptB protein catalyzes PE modification of the inner core of LPS, using phosphatidylethanolamine as a donor substrate [17]. Many Gram-negative species possess EptB-related proteins implicated in LPS/LOS PE modification including *Salmonella enterica* CptA [18] and PmrC [3] as well as LptA, Lpt3 and Lpt6 [19] from *N. meningitidis*. The neisserial PptA protein (phospho-form transferase A) is also structurally related to EptB and is necessary for modification of *N. gonorrhoeae* proteins with both PE and PC [14], [20]. In *N. meningitidis,* PptA is implicated in PC modification of PilE and high-frequency frame-shifting events within *pptA* correlate with phase (on-off) variation of the PilE PC epitope [11].

The structural relatedness between PptA and EptB and other PE transferases utilizing phosphatidylethanolamine as a donor strongly suggests that PptA employs a similar mode of action. It might be assumed that PptA differentially uses both phosphatidylethanolamine and phosphatidylcholine as precursors. However, the latter phospholipid has been only documented once in *N. gonorrhoeae* [21], while a follow up study of gonococcal phospholipids failed to document its presence [22]. Although phosphatidylcholine is found in diverse groups of bacteria, there are no established instances in which its head group serves as a precursor for phosphoform modification of LPS/LOS or proteins.

So far, two pathways for phosphatidylcholine synthesis are established in bacteria: one where endogenous phosphatidylethanolamine undergoes methylation by phospholipid *N*-methyltransferases and another in which it is synthesized via direct condensation of exogenous choline with CDP-diacylglyceride mediated by phosphatidylcholine synthase [23]. The latter process is responsible for PC modifications of both LPS/LOS and teichoic/lipoteichoic acids and utilizes enzymes encoded by the *lic* genes [4], [9], [24]. Although present in commensal neisseria species where they are involved in LOS modification, *lic* genes are absent in both *N. gonorrhoeae* and *N. meningitidis* [9]. Thus far, the mechanism by which phosphatidylcholine is produced in the pathogenic neisseria remains unknown and neither phospholipid *N*-methyltransferases nor phosphatidylcholine synthase orthologs are readily identifiable within neisserial genome sequences.

Another confounding feature of PptA-mediated protein phospho-form modification relates to the variability with which it is observed in gonococci. Under standard laboratory growth conditions gonococcal phospho-form modified proteins carry PE. However, under other growth conditions and in some genetic backgrounds a subset of these modification sites may instead carry PC [10], [14], [20]. For example, a mixture of PE and PC modified PilE was observed in a mutant carrying a null allele of *pilV* (encoding a Tfp pilin like protein that modifies Tfp-related functions) while PilE from the wild-type background carried only PE. The primary site of phospho-form modification is serine 68 of the mature gonococcal PilE protein, which is in the immediate vicinity of the glycosylation site at serine 63. In addition, there is a second phopho-form modification site at serine 156, in the C-terminus of PilE, that is only partially utilized. However, in the *pilV* null mutant the PE modification at serine 156 is more common [25]. Furthermore, it has been shown that the glycosylated lipoproteins Ngo1043 and Ngo1237 are modified with PE in a wild-type background while PC was detected in a background defective in broad spectrum *O*-linked protein glycosylation [14]. The phospho-form modification of the lipoproteins was also *pptA* dependent [14]. However, in contrast to what was observed for PilE, PilV status had no effect on phospho-form microheterogeneity of Ngo1043 and Ngo1237 [14]. It is therefore clear that the phospho-form modification status may be modulated by ancillary factors in a protein substrate-specific fashion.

To gain insights into the molecular basis for the PE to PC switch, we sought to identify factors other than PilV involved in the pilus biogenesis processes that modulate PilE phospho-form modification status. Here, we present new data establishing a connection between differential PC modification and the integrity of the Tfp biogenesis pathway. Our data also provide further insight into interplay between *O*-linked protein glycosylation and phospho-form modification status. Finally, we present data to support a model in which stepwise PE methylation is involved in PC phospho-form modification.

## Experimental Procedures

### Bacterial strains and culture conditions

The bacterial strains used in this study were derived from the MS11 background and are described in Table S1. GenBank accession numbers refer to MS11 sequences where appropriate and available. Strains were grown in conventional liquid GC medium or on GC medium plates [26], except that Thiotone E Peptone was replaced by Proteose Peptone No. 3 (Difco). When needed, antibiotics were used in the following concentrations: kanamycin (50 µg/ml), chloramphenicol (30 µg/ml) and erythromycin (8 µg/ml).

Wild-type and altered *pilE* alleles (E5L, E5V [27], G1S [28], AAM [27], I4T, V9M, A20T, W109S [29], AAM/c6His, E5K/c6His [30]) were ectopically expressed from the *iga* locus and introduced into the genomes of strain 4/3/1 and KS645 by transformation with genomic DNA and selection for erythromycin resistance. The *pilG* (*EEZ48662.1*) mutation was introduced by transformation with plasmid p12-DUS [31] and selection for erythromycin resistant transformants. Null mutants in *pptA* (EEZ48222.1) (*pptA::kan* [20]), *comP* (EEZ48527.1) (*comP::*m-Tn*erm23*)[32]), *pilD* (*EEZ48663.1*) (*pilD::*m-Tn3*erm* [26]), *pilP* (*EEZ47066.1*) (*pilP::*m-Tn*cm* [29]), *pilQ* (*EEZ47065.1*) (*pilQ::*m-Tn*cm21* [33]), *pilT* (*EEZ49064.1*) (*pilT::*m*-*Tn*cm17* [29]), *pilU* (*pilU*::m-Tn*3erm* [34]), *pilF* (*EEZ48666.1*) (*pilF::cm* [26]), *pilV* (*EEZ48312.1*) (*pilV::kan* [35]), *pilC2* (*EEZ47021.1*) (*pilC2::cm* [36]), *pilH (EEZ47439.1)* (*pilH::cm*), *pilI (EEZ47440.1) (pilI::cm)*, *pilJ (EEZ47441.1)* (*pilJ::cm*), *pilK (EEZ47442.1)* (*pilK::cm*), *pilI (EEZ47443.1)* (*pill::cm*) [37], *pglC* (*EEZ47055.1*) (*pglC::kan* [10]), and *pglE* (where the *pglE*_ON_ phase–on sequence [38] is identical to the native FA1090 sequence *NGO0207*/AAW88960) were introduced into various strain backgrounds by transformation as previously described [14], [38], [39]. Mutants were verified by PCR and/or immunoblotting with appropriate antibodies.

### Construction of a strain carrying three copies of *pilE*

A SacI fragment from pPilE2 containing the entire *pilE* ORF and 5′ promoter sequences was cloned into p2/16/1 digested with SacI [40], [41]. Among a number of clones screened by PCR and sequencing for insertion of the *pilE* ORF, one turned out to contain two copies of the SacI fragment positioned as a direct tandem repeat. The two linked *pilE* loci were introduced into the *iga* locus of the wild-type strain N400 using transformation with the p2/16/1 derivative (KP26) and selection on GC agar plates containing erythromycin. The resulting strain KS647 (3x*pilE*) thus contained three copies of the *pilE* locus.

### Pilus purification

Pili were purified by the ammonium sulfate procedure as previously described [40]. Briefly, cells were resuspended and vortexed in 0.15 mM ethanolamine buffer (pH 10.2). Cells shear depleted of pili were removed by centrifugation at 16.000 g for 5 minutes, and pili were precipitated from the suspension by addition of 1/10 volume of saturated ammonium sulfate solution. Pili were subsequently washed twice in 0.05 mM Tris buffered saline (pH 8).

### SDS-PAGE and immunoblotting

Whole cell lysates for immunoblotting were made from equal numbers of cells grown overnight on GC plates. Lysates of cells shear depleted of pili were made from the pellet recovered in the pilus purification procedure that was washed once in 1.15 mM ethanolamine buffer (pH 10.2) and diluted relative to the OD/cell number used for the original pilus purification. Procedures for SDS-PAGE and immunoblotting are described in [26], [27]. Pilin was detected using the α-pilin rabbit polyclonal antibody at 1∶1000 dilution. These antibodies were raised against the PilE-derived synthetic peptide ^51^KSAVTEYYLNHGKWPENNTSA^71^ (the peptide is numbered according to the unprocessed full length protein) [27]. PC was detected using the mouse monoclonal TEPC-15 antibody (Sigma) at 1∶750 dilution. The *N. gonorrhoeae* Ac-Gal_2_-diNAcBac trisaccharide was detected using the rabbit monoclonal npg3 antibody [39].

### Sample preparation and infusion MS analysis of intact PilE protein

PilE protein samples were cleaned with a methanol/chloroform procedure as described [1]. The pellet was dried in an inverted tube for 5 minutes before sample was dissolved in 100 µl of 10% MeOH, 70% formic acid, acetonitrile 1∶1∶3 (v/v/v). Samples were subjected to mass spectrometric analysis or frozen at −80°C. All data were acquired on a LTQ OrbitrapXL mass spectrometer (Thermo Electron, Bremen) operated by Xcalibur 2.0, in the positive ion mode. The LTQ OrbitrapXL mass spectrometer was calibrated (proteomass LTQ/FT-hybrid Cal Mix, Supelco) and tuned using the ion at *m/z* 1021 prior to PilE analysis. Mass spectrometric analyse of PilE was performed with common parameter settings of: spray voltage at 3200 V, capillary temperature at 275°C, capillary voltage of 30 V, tube lens voltage of 110 V and sheath gas flow of 8 a.u. Sample solutions were infused into the ESI source at a flow rate of 7 µl/min by a syringe. Protein mass spectra were acquired at a resolution of 60000 at *m/z* 400. Protein masses were determined by deconvolution using the integrated Xcalibur 2.0 extract algorithm. Masses of unmodified and modified proteins were determined from calculated theoretical masses, mass differences and previous work [25]. All amino acids are numbered according to the unprocessed full length protein.

### In-gel protein digestion

Coomassie stained gel slices of purified PilE were washed and destained as previously described [25] except without alkylation and reduction. Digestion steps with trypsin (Sigma) at 37°C over night were performed as described previously [25]. Dried samples were frozen at −80°C and redissolved in 0.1% formic acid prior to liquid chromatographic tandem mass spectrometric (LC-MS^2^) analyses.

### Reverse phase LC- MS^2^ analysis of proteolytic peptides

Nanoflow LC-MS and MS^2^ analyses (nano-LC-MS^2^) of proteolytic peptides were performed using an Agilent 1200 series capillary HPLC system with a corresponding autosampler, column heater, and integrated switching valve coupled via a nanoelectrospray ion source to a LTQ-Orbitrap mass spectrometer (Thermo Fisher Scientific, Bremen, Germany) as previously described [14] with the following modifications: only a gradient over 60 minutes from 5% to 55% of acetonitrile with 0.1% formic acid employing a flow rate of 0.2 µl min^−1^ was used.

A 5 µl peptide sample was injected onto the extraction column (Zorbax 300 SB-C18 of 5 µm particle size, 5 by 0.3 mm (Agilent)) and then washed with a mobile phase of 0.1% formic acid and 3% acetonitrile by the capillary pump with a flow rate of 4 µl min^−1^. The peptides were eluted from the extraction column in the back-flush mode onto the C_18_ reverse phase column (0.075×150 mm, GlycproSIL C18–80 Å, Glycpromass, Stove, Germany). The mobile phase consisted of acetonitrile and MS grade water, both containing 0.1% formic acid. LC separation was achieved with a gradient from 5% to 55% of acetonitrile with 0.1% formic acid over 60 minutes employing a flow rate of 0.2 µl min^−1^. Nanospray ionization was achieved by applying a 1.2 kV voltage between an 8 µm diameter emitter (PicoTip Emitter, New Objective, Woburn, MA) and the capillary entrance of the Orbitrap. Mass spectra were acquired in the positive-ion mode applying a data-dependent automatic switch between survey scan and MS^2^ acquisition on the Thermo Scientific LTQ OrbitrapXL mass spectrometer operated by Xcalibur 2.0. The six most intense ions from one full survey scan at a resolution of 30000 at *m/z* 400 were fragmented by higher-energy C-trap dissociation (HCD) with the first *m/z* fixed at 120 at a resolution of 7500 at *m/z* 400. All Orbitrap analyses were performed with the lock mass option (lock mass set at *m/z* 445,120024 [42]) for internal calibration. The ion selection threshold was 500 counts and selected fragment ions were dynamically excluded for 180 seconds.

## Results

### Mutations that disrupt pilus assembly impact on PC modification of PilE

Previous analyses of PilE phospho-form modifications have been made on strains expressing pili on the cell surface. In the course of our studies of mutants failing to express surface localized Tfp, we have occasionally observed alterations in the relative mobility of PilE analogous to that seen in *pilV* null backgrounds that might be indicative of altered PTMs. Therefore, we examined this possibility in more detail using a panel of congenic strains carrying null alleles of *pilD* (encoding the prepilin peptidase), *pilF* (the assembly ATPase [26]), *pilQ* (the outer membrane channel/secretin [43]), *pilP* (an inner membrane lipoprotein recquired for transformation [33]) and *pilG* (required for transformation [44]). These mutants are all unable to express pili on the cell surface. But, in contrast to the other mutants that fail to assemble pili under any circumstance, the *pilQ* mutant assembles pili that are retained in the periplasm when the PilT retraction ATPase is absent [45]. PilE from whole cell extracts of all these mutants demonstrated strong PptA-dependent reactivity with the monoclonal PC recognizing antibody TEPC-15, whereas the wild-type background, which expressed normal levels of surface localized pili, showed no reactivity (Fig 1B). PilE carrying an alanine substitution at residue 68 (a *pilE*_S68A_ background) abolished the TEPC-15 reactivity in all backgrounds suggesting that PC modification occurred on serine 68, consistent with previous results derived from analysis of PC modification in *pilV* mutants [25]. Together, these results suggest that differential PC modification of PilE can be associated with disruption of Tfp expression.

### Impact of effectors of Tfp dynamics and function on PC modification of PilE

**Figure 2:**
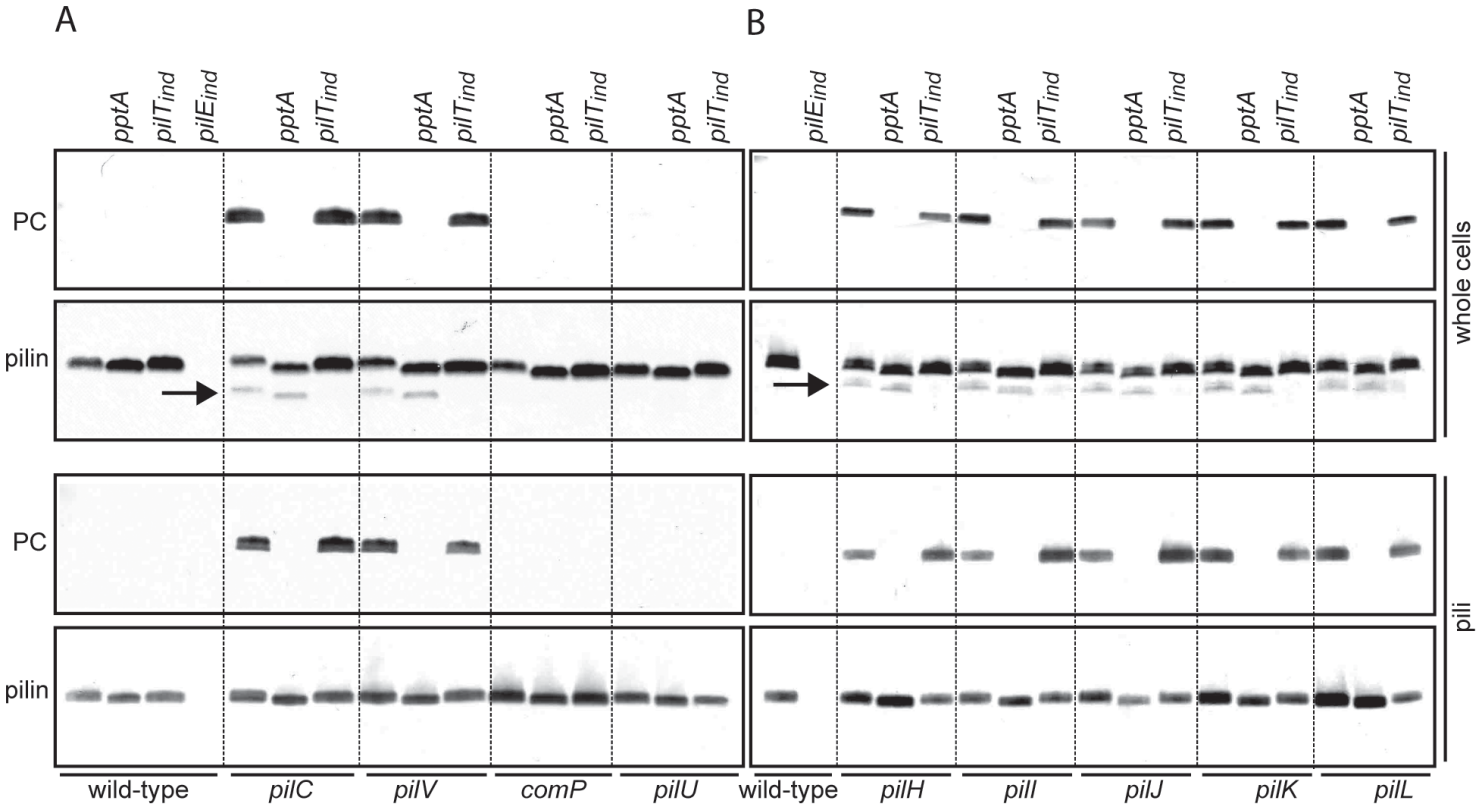
Lack of pilus associated proteins leads to PptA-dependent PC modification of PilE. Immunoblot analysis of whole cell lysates of equal numbers of cells and of equal amounts of protein from purified pili. The antibodies used were the PC recognizing TEPC-15 and the PilE peptide specific antibody α-pilin. Strains used were in A) wild-type (N400), *pptA* (KS9), *pilT*_ind_ (12/9/1), *pilE*_ind_ (MW24), *pilC* (KS787), *pilC pptA* (KS788), *pilC pilT*_ind_ (KS789), *pilV* (KS790), *pilV pptA* (KS10), *pilV pilT*_ind_ (KS791), *comP* (KS792), *comP pptA* (KS793), *comP pilT*_ind_ (KS794), *pilU* (KS795), *pilU pptA* (KS796), *pilU pilT*_ind_ (KS798) and in B) wild-type (N400), *pilE*_ind_ (KS786), *pilH* (KS799), *pilH pptA* (KS800), *pilH pilT*_ind_ (KS801), *pilI* (KS802), *pilI pptA* (KS803), *pilI pilT*_ind_ (KS804), *pilJ* (KS805), *pilJ pptA* (KS806), *pilJ pilT*_ind_ (KS807), *pilK* (KS808), *pilK pptA* (KS809), *pilK pilT*_ind_ (KS810), *pilL* (KS811), *pilL pptA* (KS812) and *pilL pilT*_ind_ (KS813). The faster migrating protein band below pilin is S-pilin (indicated by an arrow), a proteolytic degradation product of PilE that is a correlate of type IV pilus biogenesis defects and which requires *pilT* expression [36]. The strains were grown on standard GC plates without inducer such that the *pilT*_ind_ and *pilE*_ind_ loci were not expressed. All samples on each blot were run on the same gel and the dotted lines were introduced as guidance facilitating evaluation of the data. Results representative of at least three different experiments are shown.

To gain further insights into factors affecting PilE phospho-form modification, we next examined pilus-associated factors that impact Tfp dynamics and function but are dispensable for assembly of Tfp [35], [37]. Such mutants fall into two classes: one in which Tfp assembly is maintained at high levels while Tfp-associated functions are disrupted, and another in which Tfp assembly is significantly reduced. For the latter class Tfp assembly defects can be suppressed by loss-of-function mutations in PilT, the retraction ATPase, but despite their piliated phenotype, these suppressor mutants are defective in Tfp-associated functions [36], [37]. From the first class of mutants examined were those lacking the pilin-like proteins PilV [35], ComP which expression is essential for transformation [32], as well as PilU, a second ATPase influencing Tfp-associated autoagglutination [34]. We confirmed the PptA-dependent PC modification associated with the *pilV* mutation but observed no TEPC-15 reactivity in the other two mutants or the wild-type background (Fig. 2). We next tested six mutants from the second class including those lacking the Tfp associated adhesin PilC [46] and null mutants individually lacking the pilin-like proteins PilH, PilI, PilJ, PilK and PilL (encoded within the *pilH-L* locus). These are all required for expression of normal levels of piliation but their individual roles in pilus biogenesis are unknown [37]. In these mutants PilE in whole cell lysates and purified pili showed PptA-dependent PC modification. This reactivity correlated with expression of S-pilin, a soluble smaller pilin that is a cleavage product of the mature pilin protein and that has been correlated with defects in pilus assembly [28], [47]. However, PC reactivity was not observed for S-pilin. Conditional repression by *pilT* in the *pilH-L* and *pilC* mutants restored high-level Tfp expression but did not result in any discernible difference in PC reactivity of PilE compared to the *pilT* wild-type background (Fig. 2). Thus, altered phospho-form modification profiles in the *pilC* and *pilH, I, J, K* and *L* mutants phenocopy that seen in the *pilV* background.

### Influence of *pilE* missense mutations on phospho-form modifications

**Figure 3:**
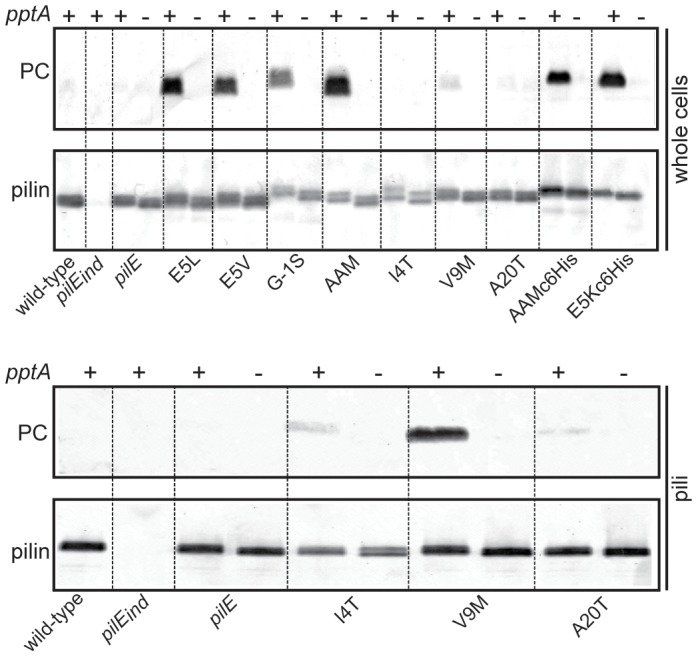
Mutations in *pilE* that perturb assembly of Tfp lead to PC modification of PilE. Immunoblot of whole cell lysates of equal numbers of cells and of equal amounts of protein from purified pili. The antibodies used were the TEPC-15 antibody and the PilE peptide specific α-pilin antibody. – denotes a null allele and + denotes a wild-type allele of *pptA*. Strains used were wild-type (N400), *pilE*_ind_ (4/3/1), *iga::pilE* (KS130), *iga::pilE pptA* (KS813), *iga::pilE*_E5L_ (KS814), and *iga::pilE*_E5L_ *pptA* (KS815) *iga::pilE*_E5V_ (KS816), and *iga::pilE*_E5V_ *pptA* (KS817), *iga::pilE*_G1S_ (KS818), and *iga::pilE*_G1S_ *pptA* (KS819), *iga::pilE*_AAM38-40_ (KS769), and *iga::pilE*_AAM38-40_ *pptA* (KS821), *iga::pilE*_I4T_ (KS722), and *iga::pilE*_I4T_ *pptA* (KS723), *iga::pilE*_V9M_ (KS724), and *iga::pilE*_V9M_ *pptA* (KS774), *iga::pilE*_A20T_ (KS775), and *iga::pilE*_A20T_ *pptA* (KS776), *iga::pilE*_AAM38-40His_ (KS525), *iga::pilE*_AAM38-40His_ *pptA* (KS781), *iga::pilE*_E5KHis_ (KS784), and *iga::pilE*_E5KHis_ *pptA* (KS820). The strains were grown on standard GC plates without inducer such that *pilE*_ind_ was not expressed. All samples on each blot were run on the same gel and the dotted lines were introduced as guidance facilitating evaluation of the data. Results representative of at least three different experiments are shown.

Our results suggest that block or delay in pilus assembly leads to PC modification of pilin. However, it is unclear whether the PC modification only relates to dysfunction of the pilus biogenesis machinery or could also be induced by alteration of the pilin molecule itself. To investigate this, we next sought to find out if mutations within *pilE* that are known to impede pilus assembly or affect pilus morphology and/or associated functions also lead to PC modification. Various *pilE* mutants were expressed from the *igA* locus in a background in which the endogenous pilin locus was under the control of an IPTG derepressible promoter [40]. No IPTG was added to the cultures and thus, the endogenous pilin locus remained silent. We tested the *pilE*_E5L_, *pilE*_E5V_ and *pilE*_E5Kc6His_ mutants which carry substitutions at the highly conserved charged residue glutamine +5 (E5) [27], [30], [48]-[50], the *pilE*_G-1S_ mutant which encodes a glycine to serine substitution at position -1 relative to the mature protein [28], the PilE_AAM38-40_ triple substitution mutant which produces rare short Tfp and is blocked in S-pilin production [27], and the *pilE*_I4T_, *pilE*_V9M_ and *pilE*_A20T_ mutants which carry alterations in the conserved amino terminal domain of PilE. The E5L, E5V, G-1S, AAMc6His and E5Kc6His mutations have all been shown to preclude Tfp expression and associated functions [28], [30], [48]-[51]. The I4T, V9M, and A20T mutants all express Tfp at levels similar to the wild-type strain but exhibit a non-aggregating phenotype and reduced ability to adhere to a human epithelial cell line [29]. Moreover, pilin from these mutants assembles into pili that are morphologically distinct from pili of the wild-type strain [29]. As shown in Fig. 3 all mutants devoid of pilus expression demonstrated strong *pptA*-dependent PC reactivity of PilE. Furthermore, the *pilE*_AAM_ mutant that makes few and short pili was also among those with strong PC reactivity. Far more reduced PC reactivity was seen in both whole cells and purified pili from the 3 missense mutants *pilE*_I4T_, *pilE*_V9M_, and *pilE*_A20T_. For these missense mutants, the PC reactivity appeared stronger in purified pili but this was probably due to a higher concentration of PilE in extracts of isolated pili. Together, these results suggest a correlation between severity of assembly defect and degree of PC reactivity in the *pilE* mutants.

### Overexpression of *pilE* is associated with PC modification

**Figure 4:**
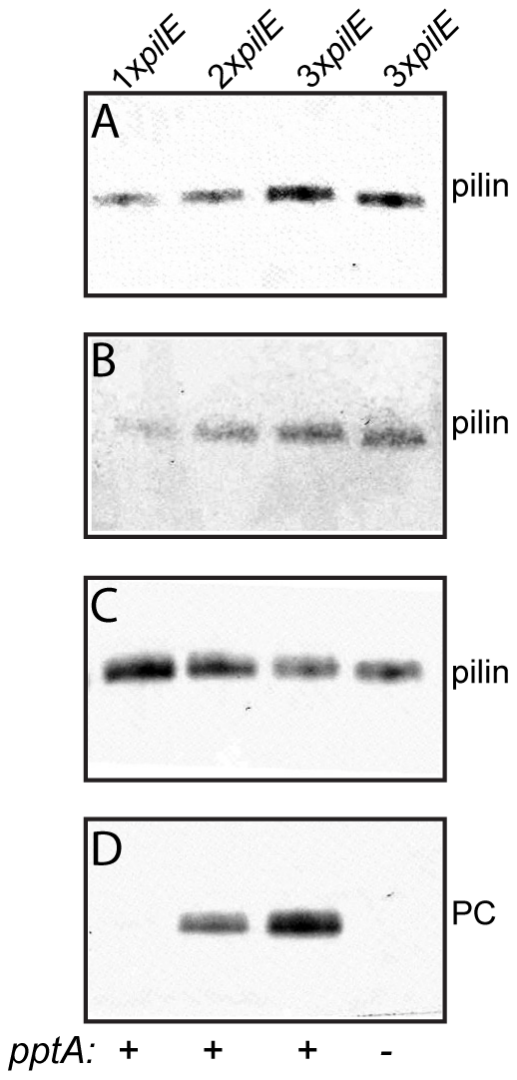
Overexpression of PilE results in increased PC-modification. Shown are immunoblot analyses of cell lysates made from equal numbers of cells of A) whole cells and B) cells recovered following shear depletion of pili. In whole cell lysates C) and D) the 2x*pilE* and 3x*pilE* strains were diluted 1∶1 and 1∶2 to account for the increased amount of PilE. The antibodies used were the PC recognizing antibody TEPC-15 and the PilE recognizing α-pilin antibody. – denotes a null allele and + denotes a wild-type allele of *pptA*. The strains used were the wild-type (N400) expressing one copy of *pilE*, 2x*pilE* (*iga*::*pilE*, i.e. a wild-type background expressing two copies of *pilE*) (KS646), 3x*pilE* (*iga*::2x*pilE*, i.e. a wild-type background expressing three copies of *pilE*) (KS647), and 3x*pilE pptA* (*pptA iga*::2x*pilE*, i.e. a *pptA* background expressing three copies of *pilE*) (KS653). Results representative of at least three different experiments are shown.

Tfp undergo bidirectional remodeling of pilin subunits between an integral inner membrane state and a multimeric, filamentous state. Alteration in this remodeling is a common feature of many of the mutants displaying altered phospho-form modification. We hypothesized that increasing the amount of PilE in a background where other Tfp expression factors were constant might lead to accumulation of pilin in the inner membrane state and in turn lead to PC modification. To address this directly, we constructed strains carrying either one or two identical additional copies of *pilE* at an ectopic site. Doubling or tripling *pilE* copy number led to increased levels of PilE in whole cells and increased levels of intracellular pilin in cells depleted of pili by shear fractioning (Fig. 4A and B, respectively). Analyses of whole cell lysates that had been adjusted to account for the increased *pilE* content demonstrated PptA-dependent PC modification in the 2x*pilE* and 3x*pilE* backgrounds and showed that the levels of PC modification were greater in the latter background (Fig 4C and D). Thus, the magnitude of PC modification was manifest in a *pilE* dose-dependent fashion, and correlated with increased level of intracellular pilin.

### PilE glycosylation status influences its phospho-form modification status

The *N. gonorrhoeae* general *O*-linked protein glycosylation system targets at least 19 structurally and functionally distinct proteins, of which PilE is the most abundantly expressed [14], [52]. The glycosylation process takes place at the inner membrane where a lipid-linked diNAcBac monosaccharide is produced by the PglBCD proteins and further elongation of the glycan occurs through the action of the glycosyltransferases PglA and PglE. Our strain background naturally contains a phase off version of *pglE*, and hence normally produces only a disaccharide. After elongation the lipid-linked oligosaccharide is flipped into the periplasmic space and the glycan transferred *en bloc* onto target proteins [52].

**Figure 5:**
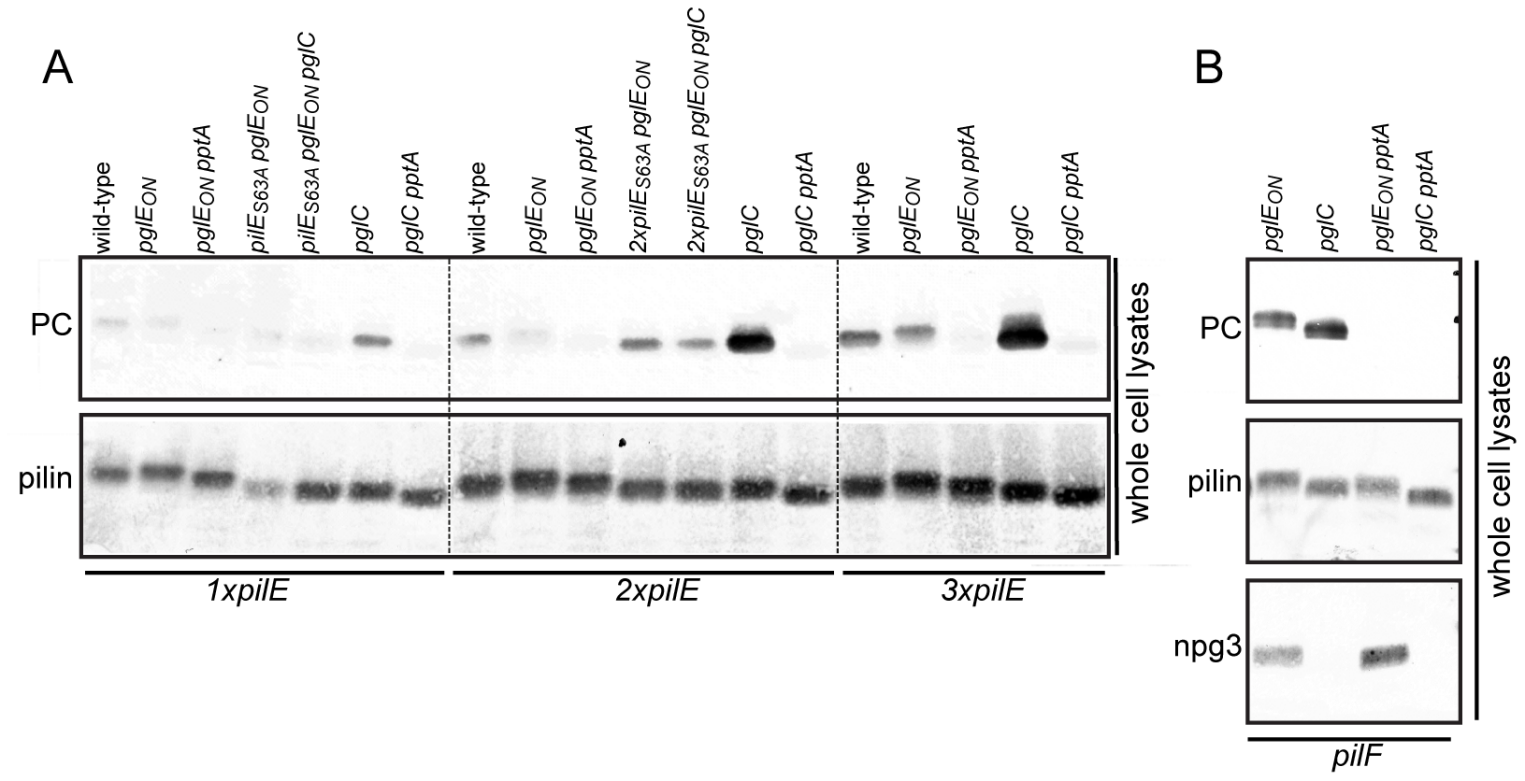
Glycosylation status affects the level of PC modification. A) Figure shows immunoblots of whole cell lysates using the PC recognizing antibody TEPC-15 and the PilE peptide specific α-pilin antibody. The samples were loaded such that each lane showed equal numbers of cells. Strains used were wild-type (N400), *pglE*_ON_ (KS142), *pglE*_ON_ *pptA* (KS651), *pilE*_S63A_ *pglE*_ON_ (KS858), *pilE*_S63A_ *pglE*_ON_ *pglC* (KS859), *pglC* (KS649), *pglC pptA* (KS652), *pglC pilT* (KS860), 2x*pilE* (KS646), 2x*pilE pglE*_ON_ (KS655), 2x*pilE pglE*_ON_ *pptA* (KS656), 2x*pilE*_S63A_ *pglE*_ON_ (KS861), 2x*pilE*_S63A_ *pglE*_ON_ *pglC* (KS862), 2x*pilE pglC* (KS657), 2x*pilE pglC pptA* (KS658), 2x*pilE pglC pilT* (KS863), 3x*pilE* (KS647), 3x*pilE pglE*_ON_ (KS654), 3x*pilE pglE*_ON_ *pptA* (KS661), 3x*pilE pglC* (KS659), 3x*pilE pglC pptA* (KS660), and 3x*pilE pglC pilT* (KS864). All samples were run on the same gel and the dotted lines were introduced as guidance facilitating evaluation of the data. Figure B) shows immunoblots of whole cell lysates made from equal numbers of cells using the PC reactive antibody TEPC-15, the α-pilin antibody, and the trisaccharide (Ac-Gal_2_-diNAcBac) specific monoclonal npg3 antibody. Strains used were *pglE*_ON_ *pilF* (KS851), *pglE*_ON_ *pilF pptA* (KS853), *pglC pilF* (KS852), and *pglC pilF pptA* (KS854). All samples on each blot were run on the same gel. Results representative of at least three different experiments are shown.

Pilin glycosylation status has been linked to subtle alterations in pilus biogenesis and related functions [30], [53]. In a recent study by Anonsen and colleagues a *pglC* null mutant (whose product is required for formation of the undecaprenyl pyrophosphate linked glycan donor) demonstrated PC modification of Ngo1043 and Ngo1237 [14]. Based on this knowledge, we wanted to assess whether protein glycosylation status also affected PC modification of PilE. We tested the wild-type background (producing the disaccharide Ac-Gal-diNAcBac), a strain carrying the *pglC* allele (not producing glycan), a strain carrying the *pglE*_ON_ allele (encoding a galactosyltransferase which adds a galactose to a pre-formed disaccharide producing the trisaccharide Ac-Gal_2_-diNAcBac), and *pilE*_S63A_ mutants, which carry a serine-to-alanine substitution at the site of glycan occupancy and, therefore, lack glycans on PilE. Moreover, to assess the effect of glycosylation in strains already expressing PC reactivity on PilE, we also tested the 2x*pilE* and 3x*pilE* strain (described above). As shown in Fig. 5A, PC modification was increased in the *pglC* background and the magnitude of the effect was again *pilE* dose-dependent. In order to distinguish whether this effect was due to altered PilE glycosylation or pleiotropic effects due to altered glycosylation status of other proteins, the effect of expressing *pilE*_S63A_ in the *pglE*_ON_ background was examined. The resulting *pilE*_S63A_ *pglE*_ON_ mutant lacked glycan on PilE but expressed trisaccharide on other glycoproteins. Consistent with a previous report, no PC reactivity was seen in the *pilE*_S63A_ *pglE*_ON_ mutant in the 1x*pilE* background [25]. In the 2x*pilE* and 3x*pilE* backgrounds the PC reactivity of the *pilE*_S63A_ *pglE*_ON_ strain was stronger than in the corresponding *pglE*_ON_ strains, suggesting that glycosylation status of PilE itself affected PC modification somewhat. However, in neither of the backgrounds did the *pilE*_S63A_ *pglE*_ON_ strains show PC reactivity that was as strong as that observed in the corresponding *pglC* mutants. This finding may relate to either the altered glycosylation status of PilE or that of other proteins of the glycoproteome. Alternatively, the serine 63 mutation may affect the recognition of the adjacent serine 68 as substrate for PC modification. To distinguish between these scenarios the *pglC* allele was introduced into the 1x*pilE*_S63A_ *pglE*_ON_ and 2x*pilE*_S63A_ *pglE*_ON_ backgrounds. The resulting *pilE*_S63A_ *pglE*_ON_ *pglC* and 2x*pilE*_S63A_ *pglE*_ON_ *pglC* strains, which lack glycan on PilE as well as on other proteins, did not demonstrate increased PC reactivity on PilE compared to the *pilE*_S63A_ *pglE*_ON_ and 2x*pilE*_S63A_ *pglE*_ON_ backgrounds. This suggested that the serine 63 mutation affected the recognition of serine 68 as an attachment site for PC modification such that PilE_S63A_ was no longer an optimal substrate for PC modification. In addition to seeing an effect of pilin glycosylation on PC modification in the *pglC* versus the *pglE*_ON_ mutants, we saw that increasing the protein-associated oligosaccharide chain length from a disaccharide to a trisaccharide was associated with diminished PC modification in the 2x*pilE pglE*_ON_ and 3x*pilE pglE*_ON_ backgrounds. This effect of glycosylation was also seen by mass spectrometry (MS) analysis of purified pili, where the 2x*pilE pglE*_ON_ background demonstrated less PC modification of PilE relative to the 2x*pilE pgl*C background. However, the peaks corresponding to the PC moiety and the acetylated trisaccharide on PilE were overlapping, resulting in overestimation of the level of PC modification in the 2x*pilE pglE*_ON_ background (data not shown).

The effects of pilin glycosylation could have three different causes: Firstly, the trisaccharide moiety could suppress the physiological event that leads to increased PC modification. Secondly, the bulkiness of the longer glycan adjacent to the PE/PC modification site could block the accessibility of the PC moiety by the PC reactive monoclonal antibodies. Thirdly, the presence of a longer glycan could alter the properties of PilE making it less optimal as a substrate for PC modification. To discriminate between these possibilities, the *pilF* mutation was introduced. As shown in Fig. 5B, the presence of trisaccharide on PilE did not suppress PC reactivity in the *pilF* mutant. This suggests that the trisaccharide modification of pilin did not grossly disturb the recognition of PilE as a substrate for PC modification or the accessibility of the PC epitope for the PC reactive antibodies. We therefore favor the scenario where the presence of glycan on PilE somehow helps suppress the physiological defect that leads to PC modification.

### MS analysis confirms altered PilE phospho-form micro- and macroheterogeneity

**Figure 6:**
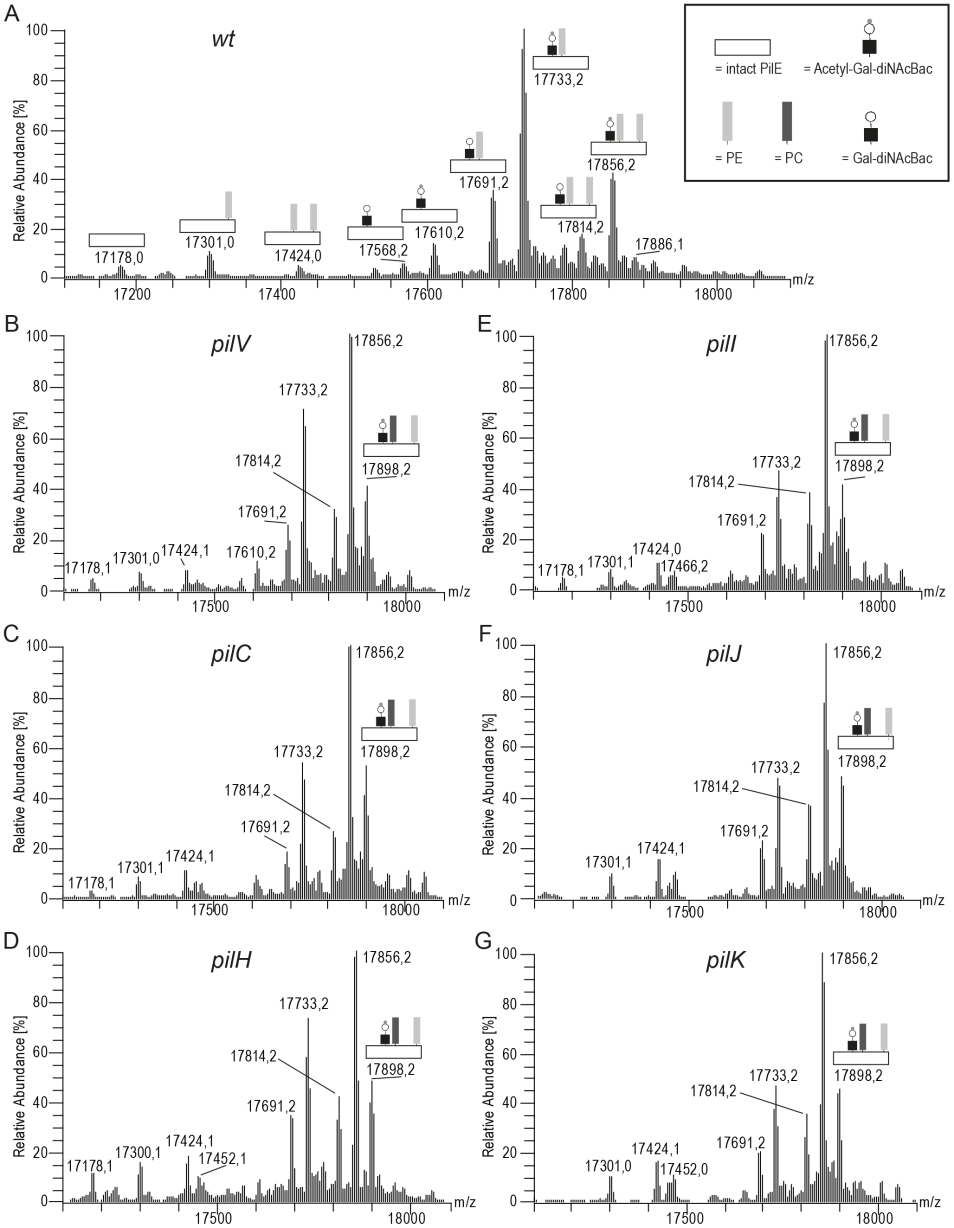
Influence of minor pilin-like proteins on PilE post-translational modifications. Deconvoluted mass spectra of intact PilE from ESI MS analysis with multiple protein forms representing species differing in glycosylation and phospho-form modifications are shown. Minor peaks and peaks connected with Na^+^ and/or K^+^ adducts in the mass spectra are not marked. PilE was isolated from A) the wild-type (N400) background, B) the *pilV* mutant (KS790), C) the *pilC* mutant (KS789), D) the *pilH* mutant (KSKS801), E) the *pilI* mutant (KS804), F) the *pilJ* mutant (KS806), and G) from the *pilK* mutant (KS810).

To confirm and extend the findings made using TEPC-15 reactivity as read-out, ‘top-down’ mass spectrometry of PilE was performed using purified Tfp from the *pilH pilT, pilI pilT, pilJ pilT, pilV pilT, pilK pilT* and *pilC2 pilT* mutants (Fig 6A–G). PE modification increases the mass of PilE by 123.0 Da whereas a PC modification will increase the mass of PilE by 165.1 Da [10], [14]. The disaccharide Ac-Gal-diNAcBac modification increases the mass of PilE by 432.2 Da. However, due to glycan microheterogeneity, mass additions to PilE of 391.1 Da (Gal-diNAcBac) and 228.1 Da (diNAcBac) can also be detected [38]. Deconvoluted mass spectra of PilE showed a broad range of protein modification forms with a peak corresponding to unmodified PilE with a mass of 17178.0 Da located to the left in the spectra [10], [14].

As shown in Fig. 6A, the most abundant species observed from the otherwise wild-type parental *pilT*_ind_ background corresponded to PilE modified with one PE moiety and one disaccharide in either its acetylated or its un-acetylated form represented by the peaks with mass 17733.2 Da and 17691.2 Da, respectively. In contrast to PilE from the parental strain, the most abundant protein form observed in all pilus effector mutants corresponded to PilE modified with two PE moieties and one disaccharide represented by the peaks with mass 17856.2 Da (acetylated disaccharide form) and 17814.2 Da (un-acetylated disaccharide form). Thus, an increase in PilE modified with two PE moieties (mass 17424.0 Da) relative to that modified with one PE moiety (mass 17301.0 Da) was observed for all mutants. In addition, peaks corresponding to PilE modified with one PC, one PE moiety and disaccharide (with a mass of 17898.2 Da) were seen in all backgrounds. Peaks corresponding to PilE modified with one PC moiety and disaccharide (mass 17775.3 Da) and PilE modified with one PE moiety and one PC moiety (mass 17466.1 Da) were also detected.

**Figure 7:**
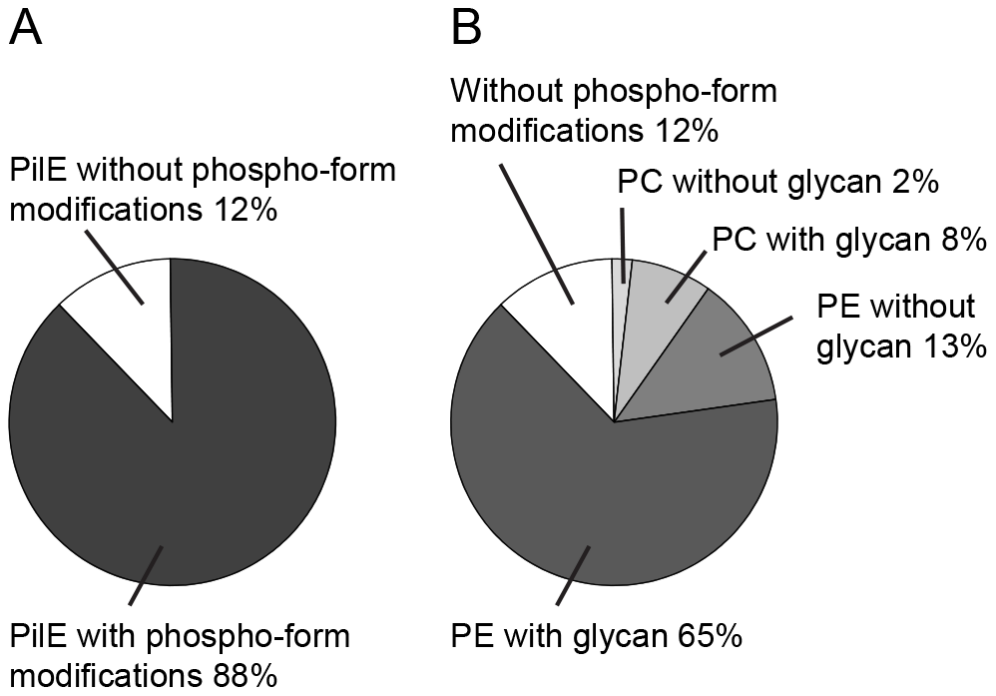
MS analysis of PTMs in purified pili from a *pilV pilT* mutant. A) Graphical representation of the relative abundance of phospho-form modified PilE compared to total PilE. B) Graphical representation of the relative abundance of various phospho-form- and glycan-modified PilE. The strain used was KS791 [35].

To investigate the ratio of PC to PE modified PilE in the effector mutants, we calculated their relative abundance in purified pili from a *pilV pilT* mutant. About 88% of total PilE was phospho-form (i.e PE and/or PC) modified and 12% of PilE was not (Fig. 7A). As seen in Fig. 6A, the majority of the PC modified pilin also carried one PE modification whereas those referred to in these calculations as PE modified carry either one or two PE. Phospho-form modified PilE could be subdivided into molecules with (78%) and without (10%) glycosylation. About 13% of PilE carried PC and glycan whereas roughly 2% of PilE carried PC but no glycan (Fig. 7B). In total, 15% of all PilE molecules carried PC that corresponded to a ratio of PC to PE modified PilE of approximately 0.2 (15%/73%).

Together these data show that PilE in all these mutants undergo alterations in phospho-form site occupancy and phospho-form microheterogeneity identical to what is previously reported for *pilV* null mutants [25]. Furthermore, the simultaneous modification of pilin molecules with both glycan and PC shows that the glycan modification of pilin did not prevent recognition of PilE as a substrate for PC modification.

### Evidence for a PE N-methyltransferase pathway for PC precursor biosynthesis

**Figure 8:**
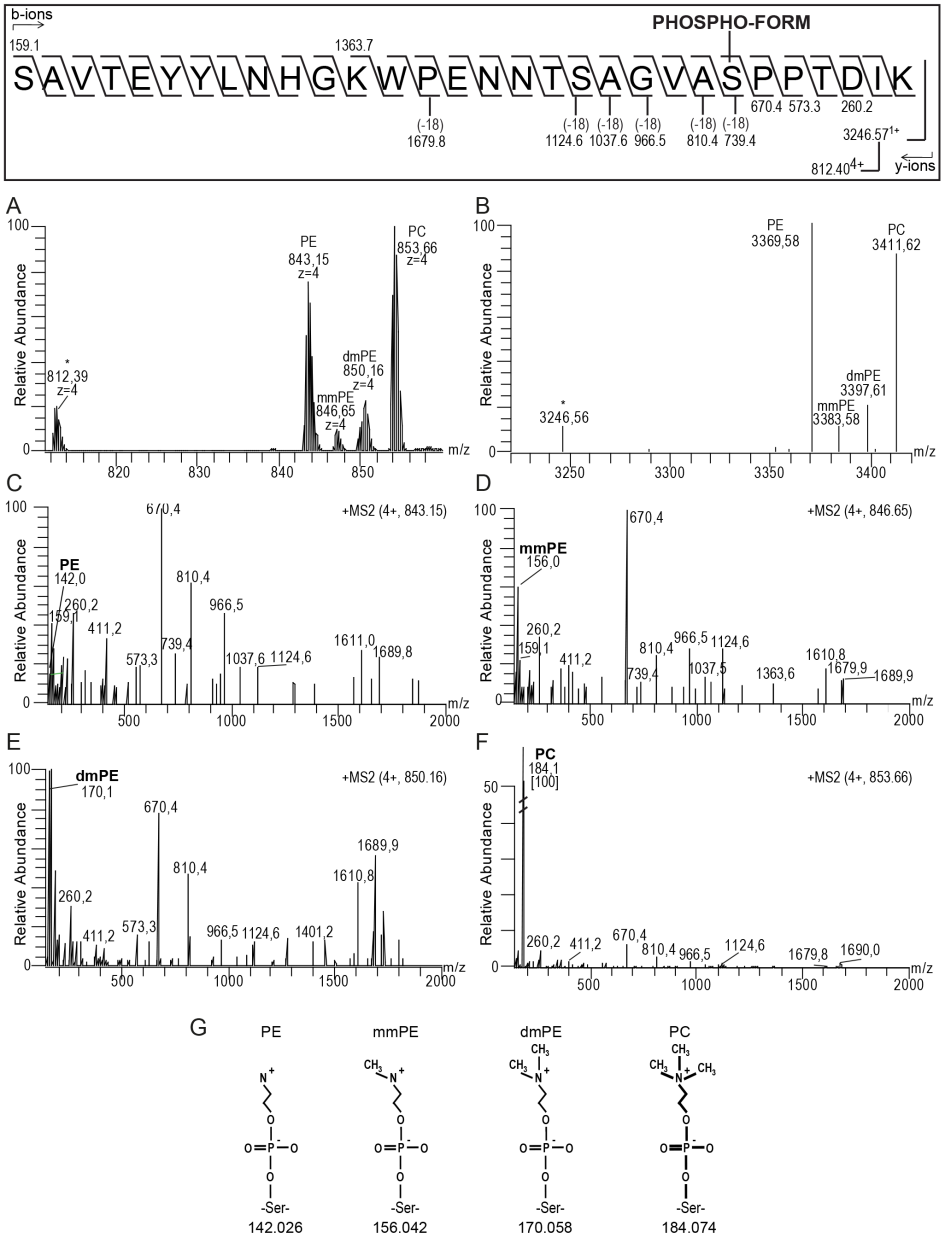
Identification of methylated PE on the PilE peptide ^52^SAVTEYYLNHGKWPENNTSAGVASPPTDIK^81^ in PilE. A) MS spectrum between m/z 800-900 of the precursor masses corresponding to the quadruply charged peptide ^52^SAVTEYYLNHGKWPENNTSAGVASPPTDIK^81^ unmodified (at *m/z* 812.90) and mass additions consistent with PE (at *m/z* 843.65), monomethylated PE (mmPE) (at *m/z* 847.15), dimethylated PE (dmPE) (at *m/z* 850.65) and PC (at *m/z* 854.16) modification. Masses are reported as monoisotopic. The asterisk denotes unmodified peptide. B) The deconvoluted mass spectrum showing the monoisotopic and monoprotonated masses of the quadruple charged ^52^SAVTEYYLNHGKWPENNTSAGVASPPTDIK^81^ peptide unmodified (at *m/z* 3246.56) and with mass additions consistent with PE (at *m/z* 3370.56), mmPE (at *m/z* 3384.58), dmPE (at *m/z* 3398.59) and PC (at *m/z* 3412.61) modification. The asterisk denotes unmodified peptide. C) MS2 HCD spectrum of the precursor peptide at *m/z* 843.65 [M+4H]^4+^ (observed monoisotopic mass of 3370.56 [M+H]^+^) confirming that peptide ^52^SAVTEYYLNHGKWPENNTSAGVASPPTDIK^81^ was modified with one PE. The reporter ion for PE at *m/z* 142.0 could be detected in the low mass area. D) MS2 HCD spectrum of the precursor peptide at *m/z* 847.15 [M+4H]^4+^ (observed monoisotopic mass of 3384.58 [M+H]^+^) confirming that peptide ^52^SAVTEYYLNHGKWPENNTSAGVASPPTDIK^81^ was modified with one mmPE. The reporter ion for mmPE at *m/z* 156.0 could be detected in the low mass area. E) MS2 HCD spectrum of the precursor peptide at *m/z* 850.65 [M+4H]^4+^ (observed monoisotopic mass of 3398.59 [M+H]^+^) confirming that peptide ^52^SAVTEYYLNHGKWPENNTSAGVASPPTDIK^81^ was modified with one dmPE. The reporter ion for dmPE at *m/z* 170.1 could be detected in the low mass area. F) MS2 HCD spectrum of the precursor peptide at *m/z* 854.16 [M+4H]^4+^ (observed monoisotopic mass of 3412.61 [M+H]^+^) confirming that peptide ^52^SAVTEYYLNHGKWPENNTSAGVASPPTDIK^81^ was modified with one PC. The reporter ion for PC at *m/z* 184.1 could be detected in the low mass area. G) The structure of PE, mmPE, dmPE and PC together with their respective reporter ion *m/z*.

In the mass spectra of PilE from the pilus biogenesis mutants, peaks indicative of the addition of one methyl group (theoretical addition of +14. 02 Da) and two methyl groups (theoretical addition of +28.04 Da) to PE were observed (Fig. 6). Peaks corresponding to addition of one and two methyl groups to PilE were not detected in mass spectra from a *pptA* deficient control background (*pptA pilV -* data not shown), indicating that the methyl groups were dependent upon the presence of a PE modification. To determine the sites of methylation, we enzymatically digested PilE from the *pglC pilV pilT* background (to avoid problems relating to the near identical masses of disaccharide acetylation and three methylation of PE) and subsequently analyzed the trypsin generated peptides by MS. The MS1 spectrum in Fig. 8A shows the quadruply charged precursor at *m/z* 812.39 corresponding to the mass of the tryptic peptide ^52^SAVTEYYLNHGKWPENNTSAGVASPPTDIK^81^ (theoretical mass 812.40 Da). Moreover, an envelope of quadruply charged precursors ions indicating additions of PE (theoretical addition of 123.01 Da) at *m/z* 843.15, of PE and one methyl group (theoretical addition of 137.03 Da) at *m/z* 846.65, of PE and two methyl groups (theoretical addition of 151.05 Da) at *m/z* 850.16 and of PC (theoretical addition of 165.06 Da) at *m/z* 853.66 to the peptide ^52^SAVTEYYLNHGKWPENNTSAGVASPPTDIK^81^ could be detected. Fig. 8B shows the deconvoluted mass spectrum of Fig 8A, i.e the same precursor masses seen in Fig. 8A presented as monoprotonated masses (calculated and adjusted for charge state). The quadruply charged precursor ion at *m/z* 843.15 (giving an observed mass of 3369.58 Da [M+H]^+^) corresponded to the peptide ^52^SAVTEYYLNHGKWPENNTSAGVASPPTDIK^81^ (theoretical monoisotopic mass 3246.57 Da) with one PE modification (Fig. 8C). Moreover, the y- and b- ions generated by fragmentation of the precursor ion at *m/z* 843.15 were consistent with the peptide ^52^SAVTEYYLNHGKWPENNTSAGVASPPTDIK^81^. In the low mass area in the fragmentation spectrum, the reporter ion at *m/z* 142.0, corresponding to (protonated) PE [10], [14], could be detected. (Fig. 8C). This demonstrates that the precursor mass at *m/z* 843.15 is the PE modified version of the peptide ^52^SAVTEYYLNHGKWPENNTSAGVASPPTDIK^81^. The quadruply charged precursor at *m/z* 846.65 (giving an observed mass of 3383.58 Da [M+H]^+^) and the quadruply charged precursor at *m/z* 850.16 (giving an observed mass of 3397,62 Da [M+H]^+^) corresponded to the peptide ^52^SAVTEYYLNHGKWPENNTSAGVASPPTDIK^81^ with one PE modification and one (Fig. 8D) or two (Fig. 8E) methyl groups, respectively. A similar pattern of y- and b- ions were generated by fragmentation of the precursors ions at *m/z* 846.65 and at *m/z* 850.16 as was seen by fragmentation of the precursor ion at *m/z* 843.15, consistent with the peptide ^52^SAVTEYYLNHGKWPENNTSAGVASPPTDIK^81^. No methylation of amino acids was detected by *de novo* sequencing of the peptides and analysis of b- and y- ions. Also, an abundant reporter ion at *m/z* 156.0, corresponding to monomethylated PE (mmPE, theoretical mass 156.04 Da) was detected in the fragmentation spectrum of the precursor ion at *m/z* 846.65 (Fig. 8D) and an abundant reporter ion at *m/z* 170.1, corresponding to dimethylated PE (dmPE, theoretical mass 170.06 Da) was detected in the fragmentation spectrum of the precursor ion at *m/z* 850.16 (Fig. 8E). The quadruply charged precursor at *m/z* 853.66 (giving an observed mass of 3411.62 Da [M+H]^+^) corresponded to the peptide ^52^SAVTEYYLNHGKWPENNTSAGVASPPTDIK^81^ with one PC modification. Moreover, in the low mass area of this spectrum, an abundant reporter ion at *m/z* 184.1, corresponding to PC was detected [10], [14] (Fig. 8F). The MS analysis therefore confirmed that the phospho-form modified peptide ^52^SAVTEYYLNHGKWPENNTSAGVASPPTDIK^81^ was modified with PE, mmPE, dmPE and PC (Fig. 8G).

Our discovery of mmPE and dmPE on a PilE phospho-form modified peptide led us to also investigate Ngo1043 for evidence of mmPE and dmPE. This was done by analyzing trypsin generated peptides from Ngo1043-His purified from a *pglC* background, i.e carrying PC, for evidence of mmPE and dmPE modified peptides [14]. As shown in Fig. S1, four precursor masses corresponding to the peptide ^66^ASAEEAVTEAK^77^ were detected with masses consistent with increasing methylation of PE. The peptide sequence of all precursor peptides was verified by *de novo* sequencing and analysis of y- and b-ions. Moreover, in the small mass area of the fragmentation spectra reporter ions of PE, mmPE, dmPE and PC were detected. The peptide ^66^ASAEEAVTEAK^77^ from Ngo1043 was therefore modified with methylated PE in a similar manner as PilE.

## Discussion

Since the discovery of phospho-form post-translational modifications in *N. gonorrhoeae*, the question regarding how PC modification is regulated has remained unanswered. Here, we characterized a defined set of mutants in which PilE undergoes PptA-dependent PC modification and that have perturbations in Tfp expression as their common denominator. These include effectors of pilus homeostasis in which Tfp dynamics are perturbed as well as *bona fide* assembly factors in which Tfp biogenesis is abolished. Analyses of PilE missense mutants also revealed a strong correlation between assembly competence and phospho-form microheterogeneity. Finally, both altered O-linked protein glycosylation and increased PilE expression were found to increase phospho-form microheterogeneity.

Our data showed that about 15% of the pilin molecules became PC modified in a Tfp effector mutant whereas the majority carried only PE. Based on this, one could argue that the major effect on phospho-form microheterogeneity could be that more of the pilins carried 2 PE moieties instead of one. However, it is worth noting that the ratio of PC to PE modified pilin also is environmentally influenced. For instance, it has been shown that growth in defined liquid medium induces PC on pilin [20]. Which of the phospho-form moieties have the greatest functional significance *in vivo* is therefore not currently clear.

How does perturbing the integrity of Tfp assembly and dynamics lead to differential phospho-form modification of PilE? As mutants of both classes show reduced or abolished piliation, they are likely to have increased intracellular concentration of the protein substrate and increased retention time of the protein substrate in the inner membrane relative to what is seen in the wild-type background. This could result in changes in membrane sublocalization and increased interaction with the enzymatic machinery responsible for PC modification, providing an explanation for our observations. This scenario could also underlie the altered phospho-form modification associated with increased PilE expression. For example, elevating the effective concentrations of PilE in the inner membrane relative to other Tfp-related proteins might create situations where the latter are limiting. Overexpressing PilE may therefore induce PC modification in much the same way as the Tfp homeostasis mutants.

Anonsen and colleagues previously reported that loss of *O*-linked protein glycosylation was associated with a switch from PE modification alone to both PE and PC in two membrane-associated glycoproteins, Ngo1043 and Ngo1237 [14]. Neither of these proteins have any known relation to pilus function or biogenesis. Another important finding from that work is that the loss of PilV, which induces PC modification of PilE, did not impact phospho-form microheterogeneity in either Ngo1043 or Ngo1237. Thus, it seems the signals provoking phospho-form microheterogeneity are intrinsic to the protein substrates themselves and result in specific or localized responses rather than global alterations in which all protein substrates are targeted. These findings argue against any model where increased PC modification is a result of increased abundance of the modifying enzyme(s) or the PC donor substrate.

Our data suggested that the glycosylation status of PilE caused increased PC modification of pilin and also in this case did the effect seem to be intrinsic to the protein itself. We have previously shown that glycosylation of PilE can impact on Tfp dynamics by effects mediated at the level of pilin subunit-subunit interactions and bidirectional remodeling of pilin between its membrane-associated and assembled states [31]. It is therefore possible that glycosylation status can induce phospho-form microheterogeneity through its effects on Tfp assembly. Alternatively, glycosylation may directly affect the efficiency with which pilin is PC modified. It is for instance possible that the glycan directly affects the pilin interaction with the enzyme machinery or the membrane sublocalization of pilin such that it co-localizes better with the PC modification machinery and the PC donor. These models could also explain the interplay between glycosylation and phospho-form modification in Ngo1043 and Ngo1237. Another possibility that has been suggested is that the glycan may sterically hinder the interaction between the PC modifying machinery and its target protein [7]. In gonococcal pilin, the glycosylation site and PC modification site are relatively close, at S63 and S68, respectively. Although we cannot completely rule out that steric hindrance has some effect, our MS data clearly show that pilin can carry both PC and glycan on the same protein molecule. In fact, the ratios of glycosylated to non-glycosylated pilin carrying PE and PC in Tfp effector mutants were very similar, suggesting that steric hindrance as a model fails to fully explain our results.

Other models that in themselves easily could explain our data regarding modification of PilE are difficult to generalize. For instance, all mutants characterized here phenocopy *pilV* null mutants with regard to PC modification and phospho-form hypermodification. Thus, an alternative explanation for our data could be that the mutations utilized here are epistatic to *pilV* and therefore preclude PilV function. It has previously been observed that hyper-modification occurs in the absence of PilV while hypo-modification occurs when PilV is overexpressed [7]. In this model PilV would inhibit PptA-dependent modification. Perhaps then, PilV might physically occlude PilE from interacting with PptA or otherwise reduce accessibility of PilE to PptA. However, it is likely that *pilV* mutants could share a common defect in the extension-retraction pathway with the other pilus related mutants. In fact, we observed here for the first time that a *pilV* null mutant expresses S-pilin, a soluble truncated form lacking the N-terminal 39 residues, and that this altered processing (but not PC modification) was suppressed in conjunction with a *pilT* null mutation. This phenotype is also observed with mutations in other effectors of pilus homeostasis, suggesting that *pilV* mutants may induce PC modification in the same way as they do.

It is important to note that an earlier study reported what was described as phase variation of the PilE PC epitope as seen by its variable detection in a panel of intrastrain pilin antigenic variants in *N. gonorrhoeae* [12]. Based on the subsequent identification of PptA as the responsible phospho-form transferase [25], and of its gene being potentially subject to high frequency, slipped strand-based frameshifting [11], it has been generally assumed that such variation was due to *pptA* phase variation. However, gonococcal strain MS11 that was used in that earlier study and that is the parent to that used by us here carries a non-phase variable *pptA* allele. Based on our findings here and the fact that different PilE antigenic variants can vary in their intrinsic assembly proficiencies, we suggest that structural PilE diversity arising via gene conversion and associated variation in assembly proclivity provides a heretofore unrecognized source for so–called phase variation of the PC epitope.

Another interesting aspect of protein targeted phospho-form micro-heterogeneity yet to be resolved relates to the source of the PC moiety. PptA is a member of the PE transferase protein family that recognizes the phospholipid head group of phosphatidylethanolamine as a donor. Accepting that phosphatidylethanolamine is the precursor to PptA-mediated modifications, the question of how and when PC is generated remains perplexing. We previously suggested one scenario in which PptA might directly utilize phosphatidylcholine as a head group donor and an alternative one in which PptA would transfer PE onto PilE that would then be transformed *in situ* into PC by PE methyltransferase(s) [20]. The phosphatidylcholine donor model is problematic due to the inability to identify phosphatidylcholine in *N. gonorrhoeae* and the lack of identifiable phosphatidylcholine synthase genes within gonococcal genome sequences. Moreover, PE (but not PC) modification was seen when PptA and PilE were co-expressed in *P. aeruginosa*, a species known to make phosphatidylcholine [20]. The second model involving methylation of PE already attached to pilin is also problematic. Specifically, the active sites of the orthologous phosphatidylethanolamine - utilizing phosphoethanolamine transferases PmrC and Cj0256 (modifying lipid A and lipid A as well as the FlgG protein respectively) are predicted to be periplasmically exposed [3], [16]. Accordingly, PE - modified PilE must a priori be localized to the periplasm. However, the methyl donor S-adenosylmethionine required for subsequent modification is unlikely to be available at this site. Thus baring retrograde re-localization of PilE to the cytoplasm (for which there is no evidence), this model is problematic as well. Whatever the case, our MS-based results clearly establish for the first time the presence of mono- and di-methylated forms of PE linked to protein. These findings do not differentiate between the scenarios but they directly implicate a methyltransferase-mediated biosynthetic pathway and corroborate data showing that PC is generated by *de novo* synthesis [20].

In conclusion, the results presented here provide insight into features affecting protein phospho-form modification and suggest that PC modification may act as a molecular marker for altered membrane protein sublocalization. Understanding how, why and when bacteria use these modifications may reveal their physiological relevance to processes operating *in vivo*.

## Supporting Information

Figure S1 **Identification of methylated PE on Ngo1043.**
(DOCX)

Table S1 **Strains used in this study.**
(DOCX)
