# Supplementary material for: Type IV Pilus Assembly Proficiency and Dynamics Influence Pilin Subunit Phospho-Form Macro- and Microheterogeneity in Neisseria gonorrhoeae
Source: PLoS One. 2014 May 5;9(5):e96419. doi: 10.1371/journal.pone.0096419 (PMC4010543; doi:10.1371/journal.pone.0096419)
Supplement: Figure S1 — Identification of methylated PE on Ngo1043. (DOCX) [file pone.0096419.s001.docx]

Figure S1.
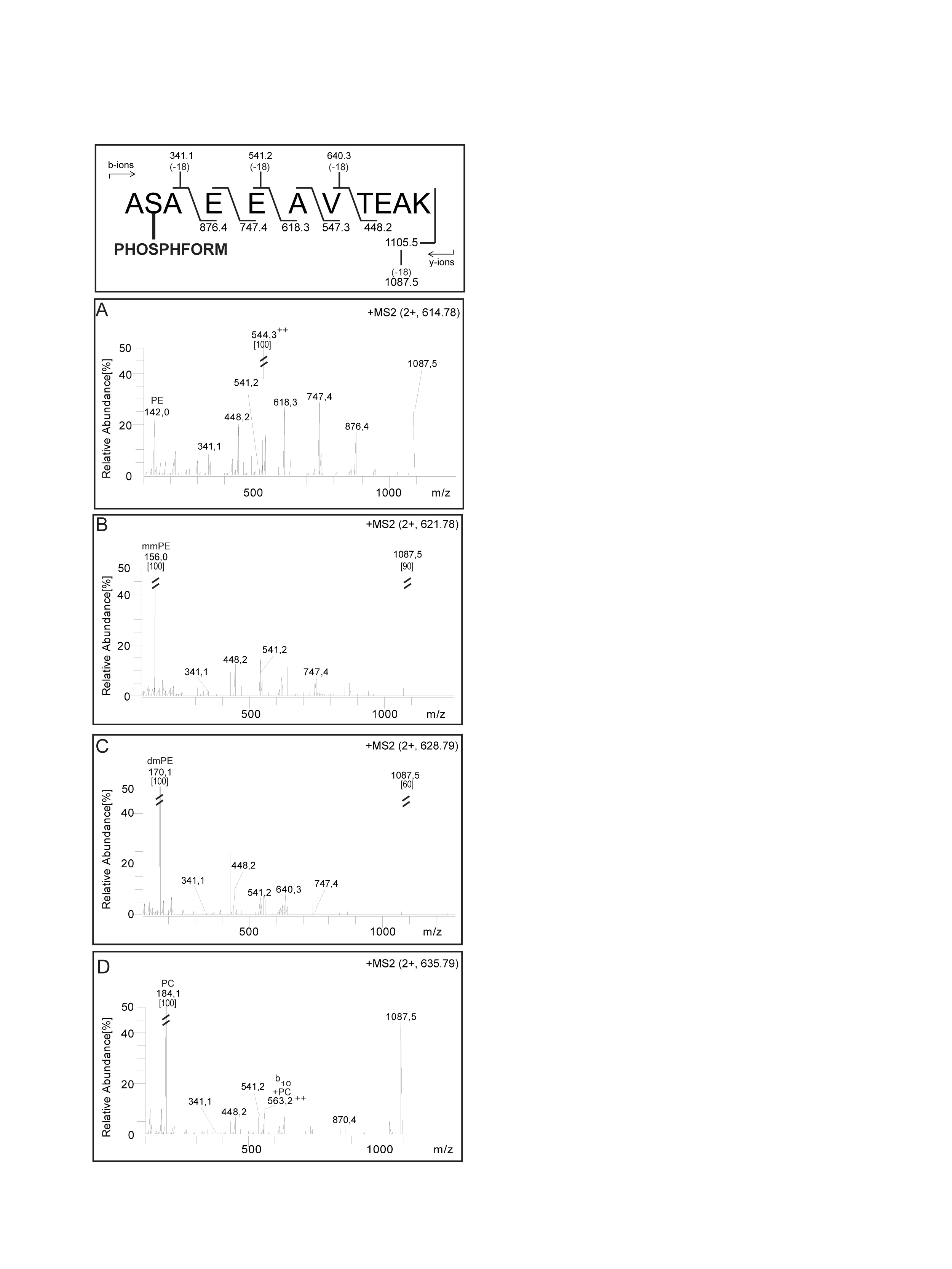


Figure S1. *Identification of methylated PE on the peptide ^66^ASAEEAVTEAK^77^ from Ngo1043*. A) MS2 HCD spectrum of the precursor peptide at *m/z* 614.78 [M+2H]^2+^ (observed monoisotopic mass of 1228.55 [M+H]^+^) confirming that peptide ^66^ASAEEAVTEAK^77^ was modified with a PE. The reporter ion for PE at *m/z* 142.0 could be detected in the low mass area. B) MS2 HCD spectrum of the precursor peptide at *m/z* 621.78 [M+2H]^2+^ (observed monoisotopic mass of 1242.55 [M+H]^+^) confirming that peptide ^66^ASAEEAVTEAK^77^ was modified with a monomethylated PE (mmPE). The reporter ion for mmPE at *m/z* 156.0 could be detected in the low mass area. C) MS2 HCD spectrum of the precursor peptide at *m/z* 628.79 [M+2H]^2+^ (observed monoisotopic mass of 1256.57 [M+H]^+^) confirming that peptide ^66^ASAEEAVTEAK^77^ was modified with a dimethylated PE (dmPE). The reporter ion for dmPE at *m/z* 170.1 could be detected in the low mass area. D) MS2 HCD spectrum of the precursor peptide at *m/z* 635.79 [M+4H]^4+^ (observed monoisotopic mass of 1270.57 [M+H]^+^) confirming that peptide ^66^ASAEEAVTEAK^77^ was modified with a PC. The reporter ion for PC at *m/z* 184.1 could be detected in the low mass area. All masses are reported as monoisotopic.
