# Supplementary material for: Type IV Pilus Assembly Proficiency and Dynamics Influence Pilin Subunit Phospho-Form Macro- and Microheterogeneity in Neisseria gonorrhoeae
Source: PLoS One. 2014 May 5;9(5):e96419. doi: 10.1371/journal.pone.0096419 (PMC4010543; doi:10.1371/journal.pone.0096419)
Supplement: Table S1 — Strains used in this study. (DOCX) [file pone.0096419.s002.docx]

**Table S1. Strains used in this study.**

| Strain | Relevant genotype | References |
| --- | --- | --- |
| 4/3/1  (MW24) | *pilE_ind_^a^* | [[1](#_ENREF_1)] |
| N400 | *recA6(tetM)^b^* | [[2](#_ENREF_2)] |
| KS9 | *recA6(tetM)^b^ pptA::kan* | [[3](#_ENREF_3)] |
| KS640 (GE68) | *recA6(tetM)^b^ pilE_S68A_* | [[4](#_ENREF_4)] |
| 12/9/1 | *recA6(Kan)^b^ pilT_ind_ (tetM)^b^* | [[1](#_ENREF_1)] |
| KS130 (MW25) | *pilE_ind_ (tetM) iga::pilE(erm)* | [[1](#_ENREF_1)] |
| KS814 | *pilE_ind_ (tetM) iga::pilE(erm) pptA::kan* | This study |
| KS814 | *pilE_ind_ (tetM) iga::pilE_E5L_(erm)* | [[5](#_ENREF_5)] |
| KS815 | *pilE_ind_ (tetM) iga::pilE_E5L_(erm) pptA::kan* | This study |
| KS816 | *pilE_ind_ (tetM) iga::pilE_E5V_(erm)* | [[5](#_ENREF_5)] |
| KS817 | *pilE_ind_ (tetM) iga::pilE_E5V_(erm) pptA::kan* | This study |
| KS818 | *pilE_ind_ (tetM) iga::pilE_G1S_ (erm)* | [[6](#_ENREF_6)] |
| KS819 | *pilE_ind_ (tetM) iga::pilE_G1S_ (erm) pptA::kan* | This study |
| KS722 | *pilE_ind_ (tetM) iga::pilE_I4T_(erm)* | This study |
| KS723 | *pilE_ind_ (tetM) iga::pilE_I4T_(erm) pptA::kan* | This study |
| KS724 | *pilE_ind_ (tetM) iga::pilE_V9M_(erm)* | This study |
| KS774 | *pilE_ind_ (tetM) iga::pilE_V9M_(erm) pptA::kan* | This study |
| KS775 | *pilE_ind_ (tetM)iga::pilE_A20T_(erm)* | This study |
| KS776 | *pilE_ind_ (tetM) iga::pilE_A20T_(erm) pptA::kan* | This study |
| KS784 | *pilE_ind_ (tetM) iga::pilE_E5Kc6His_(erm)* | [[7](#_ENREF_7)] |
| KS820 | *pilE_ind_ (tetM) iga::pilE_E5Kc6His_(erm) pptA::kan* | This study |
| KS769 | *pilE_ind_ (tetM) iga::pilE_AAM38-40_(erm)* | [[5](#_ENREF_5)] |
| KS821 | *pilE_ind_ (tetM) iga::pilE_AAM38-40_(erm) pptA::kan* | This study |
| KS525 | *pilE_ind_ (tetM) iga::pilE_AAM38-40c6His_(erm)* | [[7](#_ENREF_7)] |
| KS781 | *pilE_ind_ (tetM) iga::pilE_AAM38-40c6His_(erm) pptA::kan* | This study |
| KS792 | *recA6(tetM)^b^ comP*::mTn*erm*23 | [[8](#_ENREF_8)] |
| KS793 | *recA6(tetM)^b^ comP*::mTn*erm*23 *pptA::kan* | This study |
| KS794 | *recA6(kan)^b^ comP*::mTn*erm*23 *pilT_ind_ (tetM)^c^* | This study |
| KS787 | *recA6(tetM)^b^ pilC2::cm* | [[9](#_ENREF_9)] |
| KS788 | *recA6(tetM)^b^ pilC2::cm pptA::kan* | This study |
| KS789 | *recA6(kan)^b^ pilC2::cm pilT_ind_ (tetM)^c^* | [[9](#_ENREF_9)] |
| KS641 | *recA6(tetM)^b^ pilD::*mTn*erm* at position 525 | [[10](#_ENREF_10)] |
| KS662 | *recA6(tetM)^b^ pilD::*mTn*erm* *pptA::kan* | This study |
| KS667 | *recA6(tetM)^b^ pilE_S68A_ pilD::*mTn*erm* | This study |
| KS643 | *recA6(TetM)^b^ pilF_cat_* | [[5](#_ENREF_5)] |
| KS663 | *recA6(TetM)^b^ pilF_cat_ pptA::kan* | This study |
| KS668 | *recA6(TetM)^b^ pilE_S68A_ pilF_cat_* | This study |
| KS674 | *recA6(tetM)^b^ pilG::* mTn*erm* at position 641 | This study |
| KS673 | *recA6(tetM)^b^ pilG::* mTn*Erm* *pptA::kan* | This study |
| KS672 | *recA6(tetM)^b^ pilE_S68A_ pilG::*mTn*erm* | This study |
| KS799 | *recA6(tetM)^b^ pilH::cm* | [[9](#_ENREF_9)] |
| KS800 | *recA6(tetM)^b^ pilH::cm pptA::kan* | This study |
| KS801 | *recA6(kan)^b^ pilH::cm pilT_ind_ (tetM)^c^* | [[9](#_ENREF_9)] |
| KS802 | *recA6(tetM)^b^ pilI::cm* | [[9](#_ENREF_9)] |
| KS803 | *recA6(tetM)^b^ pilI::cm pptA::kan* | This study |
| KS804 | *recA6(tetM)^b^ pilI::cm pilT_ind_ (tetM)^c^* | [[9](#_ENREF_9)] |
| KS805 | *recA6(tetM)^b^ pilJ::cm* | [[9](#_ENREF_9)] |
| KS806 | *recA6(tetM)^b^ pilJ::cm pptA::kan* | This study |
| KS807 | *recA6(kan)^b^ pilJ pilT_ind_ (tetM)^c^* | [[9](#_ENREF_9)] |
| KS808 | *recA6(tetM)^b^ pilK::cm* | [[9](#_ENREF_9)] |
| KS809 | *recA6(tetM)^b^ pilK::cm pptA::kan* | This study |
| KS810 | *recA6(kan)^b^ pilK::cm pilT_ind_ (tetM)^c^* | [[9](#_ENREF_9)] |
| KS811 | *recA6(tetM)^b^ pilL::cm* | [[9](#_ENREF_9)] |
| KS812 | *recA6(tetM)^b^ pilL::cm pptA::kan* | This study |
| KS813 | *recA6(kan)^b^ pilL::cm pilT_ind_ (tetM)^c^* | [[9](#_ENREF_9)] |
| KS665 | *recA6(tetM)^b^ pilP::*m-Tn*cm at position 931* | [[11](#_ENREF_11)] |
| KS666 | *recA6(tetM)^b^ pilP::*m-Tn*cm pptA::kan* | This study |
| KS670 | *recA6(tetM)^b^ pilE_S68A_ pilP::*m-Tn*cm* | This study |
| KS795 | *recA6(tetM)^b^ pilU*::m-Tn*3erm* at position 2102 | [[12](#_ENREF_12)] |
| KS796 | *recA6(tetM)^b^ pilU*::m-Tn*3erm* at position 2102 *pptA::kan* | This study |
| KS798 | *recA6(kan)^b^ pilU*::m-Tn*3erm* at position 2102 *pilT_ind_ (tetM)^c^* | This study |
| KS790 | *recA6(tetM)^b^ pilV _G-1fs_* | [[13](#_ENREF_13)] |
| KS10 | *recA6(tetM)^b^ pilV_G-1fs_ pptA::kan* | [[3](#_ENREF_3)] |
| KS791 | *recA6(Kan)^b^ pilV _G-1fs_  pilT_ind_ (TetM)^c^* | [[13](#_ENREF_13)] |
| KS644 | *recA6(TetM)^b^ pilQ::m-*Tn*Cm* at position 1250 | [[11](#_ENREF_11)] |
| KS664 | *recA6(TetM)^b^ pilQ::m-*Tn*Cm* at position 1250 *pptA::Kan* | This study |
| KS669 | *recA6(TetM)^b^ pilE_S68A_ pilQ::m-*Tn*cm* at position 1250 | This study |
| KS645 | *pilE_ind_^a^ pptA::kan* | This study |
| KS646 | *recA6(TetM)^b^ iga::pilE(erm)* | [[14](#_ENREF_14)] |
| KS647 | *recA6(TetM)^b^ iga::*2x*pilE(erm)* | This study |
| KS653 | *recA6(TetM)^b^ pptA iga::*2x*pilE(erm)* | This study |
| KS659 | *recA6(TetM)^b^ pglC iga::*2x*pilE pptA(erm)* | This study |
| KS660 | *recA6(tetM)^b^ pglC pptA iga::*2x*pilE(erm)* | This study |
| KS654 | *recA6(tetM)^b^ pglE_ON_ iga::*2x*pilE(erm)* | [[15](#_ENREF_15)] |
| KS661 | *recA6(TetM)^b^ pglE_ON_ iga::*2x*pilE pptA::kan* | This study |
| KS851 | *recA6(tetM)^b^ pglE_ON_ pilF::cm* | This study |
| KS853 | *recA6(tetM)^b^ pglE_ON_ pilF::cm pptA::kan* | This study |
| KS852 | *recA6(tetM)^b^ pglC pilF::cm* | [[15](#_ENREF_15)] |
| KS854 | *recA6(tetM)^b^ pglC pilF::cm pptA::kan* | This study |
| KS649 | *recA6(tetM)^b^ pglC_fs_* | [[16](#_ENREF_16)] |
| KS855 | *recA6(tetM)^b^ ngo1717::kan* | This study |
| KS142 | *recA6(tetM)^b^ pglE_ON_* | [[15](#_ENREF_15)] |
| KS856 | *recA6(tetM)^b^ ngo1548::cm* | This study |
| KS857 | *recA6(tetM)^b^ ngo1717::kan ngo1548::cm* | This study |
| KS651 | *recA6(tetM)^b^ pglE_ON_ pptA::kan* | This study |
| KS652 | *recA6(tetM)^b^ pglC_fs_ pptA::kan* | This study |
| KS858 | *recA6(tetM)^b^ pilE_S63A_ pglE_ON_* | This study |
| KS859 | *recA6(tetM)^b^ pilE_S63A_ pglE_ON_ pglC::kan* | This study |
| KS860 | *recA6(tetM)^b^ pglC_fs_ pilT::cm2-17* | This study |
| KS655 | *recA6(tetM)^b^ pglE_ON_ igA::pilE(erm)* | This study |
| KS656 | *recA6(tetM)^b^ pglE_ON_ pptA::kan igA::pilE(erm)* | This study |
| KS861 | *recA6(tetM)^b^ pilE_S63A_ pglE_ON_ iga::pilE_S63A_* | This study |
| KS862 | *recA6(tetM)^b^ pilE_S63A_ pglE_ON_ iga::pilE_S63A_ (erm) pglC::kan* | This study |
| KS657 | *recA6(tetM)^b^ pglCfs igA::pilE(erm)* | This study |
| KS658 | *recA6(tetM)^b^ pglCfs pptA::kan igA::pilE(erm)* | This study |
| KS863 | *recA6(tetM)^b^ pglC_fs_ igA::pilE(erm) pilT::cm2-17* | This study |
| KS864 | *recA6(tetM)^b^ pglC_fs_ iga::2xpilE(erm) pilT::cm2-17* | This study |

^a^*pilE_ind_* is an IPTG-inducible allele of *pilE*.

^b^*recA6* is an IPTG-inducible allele of *recA*.

^c^*pilT_ind_* is an IPTG-inducible allele of *pilT*.

# Referenses

1. Wolfgang M, van Putten JP, Hayes SF, Dorward D, Koomey M (2000) Components and dynamics of fiber formation define a ubiquitous biogenesis pathway for bacterial pili. EMBO J 19: 6408-6418.

2. Tonjum T, Freitag NE, Namork E, Koomey M (1995) Identification and characterization of pilG, a highly conserved pilus-assembly gene in pathogenic Neisseria. Mol Microbiol 16: 451-464.

3. Naessan CL, Egge-Jacobsen W, Heiniger RW, Wolfgang MC, Aas FE, et al. (2008) Genetic and functional analyses of PptA, a phospho-form transferase targeting type IV pili in Neisseria gonorrhoeae. J Bacteriol 190: 387-400.

4. Aas FE, Egge-Jacobsen W, Winther-Larsen HC, Lovold C, Hitchen PG, et al. (2006) Neisseria gonorrhoeae type IV pili undergo multisite, hierarchical modifications with phosphoethanolamine and phosphocholine requiring an enzyme structurally related to lipopolysaccharide phosphoethanolamine transferases. J Biol Chem 281: 27712-27723.

5. Aas FE, Winther-Larsen HC, Wolfgang M, Frye S, Lovold C, et al. (2007) Substitutions in the N-terminal alpha helical spine of Neisseria gonorrhoeae pilin affect Type IV pilus assembly, dynamics and associated functions. Mol Microbiol 63: 69-85.

6. Koomey M, Bergstrom S, Blake M, Swanson J (1991) Pilin expression and processing in pilus mutants of Neisseria gonorrhoeae: critical role of Gly-1 in assembly. Mol Microbiol 5: 279-287.

7. Vik A, Aspholm M, Anonsen JH, Borud B, Roos N, et al. (2012) Insights into type IV pilus biogenesis and dynamics from genetic analysis of a C-terminally tagged pilin: a role for O-linked glycosylation. Mol Microbiol 85: 1166-1178.

8. Wolfgang M, van Putten JP, Hayes SF, Koomey M (1999) The comP locus of Neisseria gonorrhoeae encodes a type IV prepilin that is dispensable for pilus biogenesis but essential for natural transformation. Mol Microbiol 31: 1345-1357.

9. Winther-Larsen HC, Wolfgang M, Dunham S, van Putten JP, Dorward D, et al. (2005) A conserved set of pilin-like molecules controls type IV pilus dynamics and organelle-associated functions in Neisseria gonorrhoeae. Mol Microbiol 56: 903-917.

10. Freitag NE, Seifert HS, Koomey M (1995) Characterization of the pilF-pilD pilus-assembly locus of Neisseria gonorrhoeae. Mol Microbiol 16: 575-586.

11. Drake SL, Sandstedt SA, Koomey M (1997) PilP, a pilus biogenesis lipoprotein in Neisseria gonorrhoeae, affects expression of PilQ as a high-molecular-mass multimer. Mol Microbiol 23: 657-668.

12. Park HS, Wolfgang M, Koomey M (2002) Modification of type IV pilus-associated epithelial cell adherence and multicellular behavior by the PilU protein of Neisseria gonorrhoeae. Infect Immun 70: 3891-3903.

13. Winther-Larsen HC, Hegge FT, Wolfgang M, Hayes SF, van Putten JP, et al. (2001) Neisseria gonorrhoeae PilV, a type IV pilus-associated protein essential to human epithelial cell adherence. Proc Natl Acad Sci U S A 98: 15276-15281.

14. Park HS, Wolfgang M, van Putten JP, Dorward D, Hayes SF, et al. (2001) Structural alterations in a type IV pilus subunit protein result in concurrent defects in multicellular behaviour and adherence to host tissue. Mol Microbiol 42: 293-307.

15. Aas FE, Vik A, Vedde J, Koomey M, Egge-Jacobsen W (2007) Neisseria gonorrhoeae O-linked pilin glycosylation: functional analyses define both the biosynthetic pathway and glycan structure. Mol Microbiol 65: 607-624.

16. Anonsen JH, Egge-Jacobsen W, Aas FE, Borud B, Koomey M, et al. (2012) Novel protein substrates of the phospho-form modification system in Neisseria gonorrhoeae and their connection to O-linked protein glycosylation. Infect Immun 80: 22-30.
